# Macrophage Activation Syndrome Revealing Hodgkin Lymphoma: A Pediatric Case Report

**DOI:** 10.7759/cureus.82962

**Published:** 2025-04-25

**Authors:** Ahmed Hared Bouh, Omar Moussa Sougueh, Amal Miqdadi, Inssaf Al Ammari, Nouzha Dini

**Affiliations:** 1 Pediatrics, Mohammed VI International University Hospital, Mohammed VI University of Sciences and Health-UM6SS, Casablanca, MAR; 2 Pediatrics, Cheick Khalifa International University Hospital, Mohammed VI University of Sciences and Health-UM6SS, Casablanca, MAR; 3 Nuclear Medicine, Mohammed VI International University Hospital, Mohammed VI University of Sciences and Health-UM6SS, Casablanca, MAR; 4 Nuclear Medicine, Cheick Khalifa International University Hospital, Mohammed VI University of Sciences and Health-UM6SS, Casablanca, MAR

**Keywords:** adenopathy, fever, histology, hodgkin lymphoma, macrophage activation syndrome

## Abstract

Secondary macrophage activation syndrome is a severe and rare complication of infectious, autoimmune, and malignant diseases. Herein, we report the case of a 10-year-old child with this syndrome leading to the discovery of Hodgkin lymphoma. The patient, who had a medical history of cerebral palsy and ataxia-telangiectasia syndrome, was admitted for prolonged fever associated with cough, which had evolved into general health deterioration for two months prior to admission. He received multiple courses of antibiotic therapy for suspected pneumonia without clinical improvement. Clinical examination showed pallor, deteriorated general condition, high fever, asthenia, cervical adenopathy, and splenomegaly. The clinical and biological criteria led to the diagnosis of macrophage activation syndrome. Lymph node histology confirmed the diagnosis of Hodgkin lymphoma, which was staged as stage IV via positron emission tomography scan. The “vincristine, etoposide, prednisone, and doxorubicin” (OEPA) chemotherapy regimen was initiated, followed by the “cyclophosphamide, vincristine, prednisone, and dacarbazine” (COPDAC) regimen, with remission achieved both clinically and paraclinically after one cycle of each regimen.

## Introduction

Macrophage activation syndrome (MAS) is a rare and severe complication of infectious, autoimmune, and malignant diseases, and requires early diagnosis and prompt intervention. Primary MAS is a subset of hemophagocytic lymphohistiocytosis (HLH) [1]. Diagnosis of MAS is established according to the HLH-2024 criteria. MAS is an uncontrolled activation of macrophage cells, with a mortality rate of 8% to 22% [2]. The Epstein-Barr virus (EBV) is implicated in the pathogenesis of MAS and Hodgkin lymphoma [3]. MAS rarely allows the diagnosis of Hodgkin lymphoma in children. Herein, we report a pediatric case of MAS due to Hodgkin lymphoma.

## Case presentation

A 10-year-old boy was hospitalized for prolonged fever, cough, and deterioration in his general condition. The patient had no neonatal history or parental consanguinity, and was being followed since age three for ataxia-telangiectasia syndrome and cerebral palsy. The patient had no family history of malignancy or immune deficiency.

Before admission, the patient had a prolonged fever associated with a cough evolving in a context of altered general condition without signs of respiratory distress for two months. He received several pediatric consultations in the city, as well as probabilistic antibiotic therapy with unfavorable evolution, leading to admission. Clinical examination revealed pallor, high fever between 39 and 40°C, asthenia, generalized and bilateral cervical adenopathy, splenomegaly, and bilateral conjunctival telangiectasia, with the rest of the examination being unremarkable.

Blood testing revealed a hypochromic microcytic anemia, hyperferritinemia, elevated C-reactive protein (CRP), increased triglycerides, elevated alanine transaminase, hypofibrinogenemia, positive antibody IgG anti-VCA (viral capsid antigen), and decreased IgA totals, as seen in Table 1.

**Table 1:** Blood testing results MCV: mean corpuscular volume; LDH: lactate dehydrogenase; ESR: erythrocyte sedimentation rate; ALT: alanine transaminase; HIV: human immunodeficiency virus; VCA, viral capsid antigen.

Parameter	Result	Reference value
White blood cells (/mm^3^)	14,770	4.0-14.5
Red blood cells (x 10^12 ^T/L)	3.36	3.9-5.2
Hemoglobin (g/dL)	7.1	11.1-14.7
MCV (fL)	72	75-85
Hematocrit (%)	24.2	32-45
Platelets (x 10^3^/mm^3^)	726,000	166-463
Ferritin (ng/mL)	3039	15-80
Fibrinogen (g/L)	1.5	1.8-3.5
Triglyceride (g/L)	2.3	<1.5
LDH (UI/L)	355	85-230
C-reactive protein (mg/L)	202	0.1-2.8
ESR (mm)	144 at the first hour	<13
Procalcitonin (ng/mL)	0.26	<0.5
ALT (UI/L)	125	<50
Creatinine (mg/L)	4.0	4.4-6.8
Antibody IgG anti-VCA (UA/mL)	>750	<20
Antibody IgM anti-VCA (UA/mL)	<10	<40
Antibody IgG anti-EBNA (UA/mL)	<3	<20
Immunoglobulin A (g/L)	<0.02	0.5-1.7
Immunoglobulin G (g/L)	11.56	6.2-11.5
Immunoglobulin M (g/L)	1.16	0.55-1.55

The initial treatment consisted of intravenous antibiotic therapy (amoxicillin-clavulanic acid), but fever and general health deterioration persisted 72 hours after the start of treatment. The etiological workup for infectious causes, including urinalysis with culture, blood cultures, and relevant viral serologies (hepatitis C and B, human immunodeficiency virus [HIV], cytomegalovirus [CMV]), returned negative results. Subsequently, we completed the report as outlined below. Abdominal ultrasound revealed deep polyadenopathy with moderate splenomegaly. A myelogram demonstrated rich marrow and numerous macrophages activated without hematophagocytosis in relation to MAS. One week after hospitalization, a treatment with Solumedrol 2 mg/kg/day IV was initiated. The clinical course was marked by the resolution of fever.

**Figure 1:**
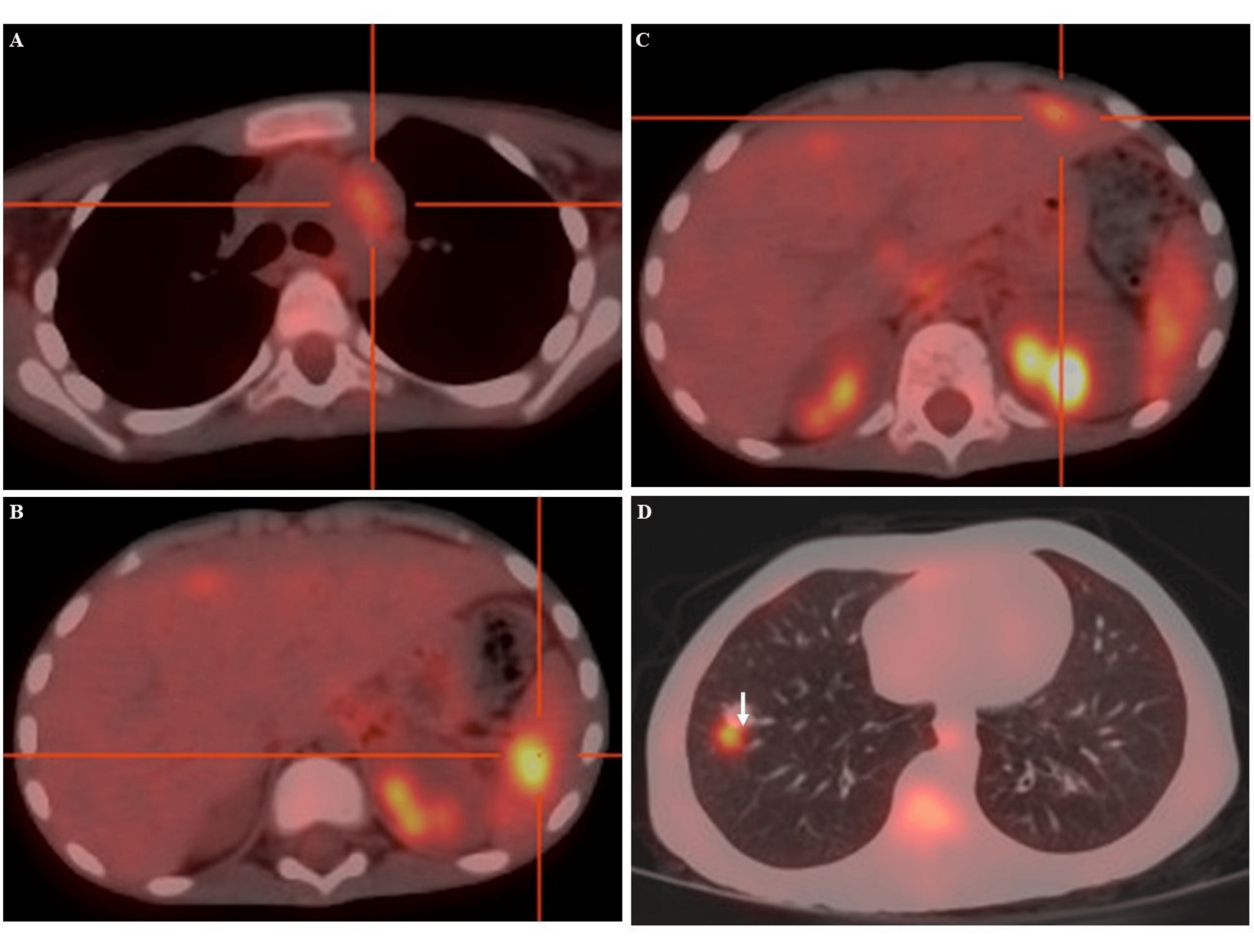
PET-CT axial views of hypermetabolic lesions on prevascular node (A), spleen (B) and liver (C) nodes, as well as a pulmonary nodule (D).

Following these results, a lymph node biopsy was performed, revealing the following: Microscopic study showed a lymph node parenchyma whose architecture is disrupted by a tumor proliferation composed of cells detaching on a granulomatous background. These tumor cells include large Hodgkin-like cells with moderately abundant cytoplasm, featuring a clear, unilobated, nucleolated nucleus with a central acidophilic nucleolus and membranous chromatin densification; other larger cells exhibit basophilic cytoplasm and multilobulated, hyperchromatic nuclei with several nucleolar aggregates. The granulomatous background is composed of lymphocytes, plasma cells, histiocytes, mast cells, and a few neutrophilic polymorphonuclear cells. The proliferation elicits a dense fibrous reaction in places. This histological appearance is consistent with Hodgkin lymphoma. Then, a PET scan also showed multiple hypermetabolic foci in the cervico-mediastinal lymph nodes, lumbo-aortic lymph nodes, hepatosplenic lymph nodes, and bones corresponding to Hodgkin lymphoma stage IV, as seen in Figure 1. 18F-FDG PET/CT was requested as part of the staging assessment. The patients fasted for 12 hours before the examination. Blood sugar level was measured prior to the injection of 18F-FDG (1.09 g/L). 18F-FDG PET-CT revealed hypermetabolic mediastinal and lumbo-aortic nodes (Figure 1A). Liver and spleen nodes were also observed (Figure 1B).

Based on all the aforementioned findings, the patient was diagnosed with MAS due to Hodgkin lymphoma with EBV infection. After one month of hospitalization in the pediatric service, he was transferred to the pediatric hematology unit. Following the oncological hematology team meeting, two consecutive chemotherapy protocols, in particular, the vincristine, etoposide, prednisone, and doxorubicin (OEPA) regimen, followed by the cyclophosphamide, vincristine, prednisone, and dacarbazine (COPDAC) regimen, were started. He received two cycles of OEPA (induction) followed by three cycles of COPDAC (consolidation) every 28 days. Remission was achieved after these cycles of chemotherapy, and his symptoms of fever and inflammatory syndrome disappeared. A follow-up PET scan showed a regression of the lymphadenopathies. Thus, he continued his validated chemotherapy protocol.

## Discussion

MAS is a rare but life-threatening condition characterized by excessive cytokine activation that can cause rapid deterioration and multi-organ failure, requiring early recognition and management. The HLH-2004 diagnostic criteria are widely used in pediatric cases, which include clinical criteria (i.e., fever and splenomegaly) and biological criteria (i.e., cytopenias, hyperferritinemia, hypertriglyceridemia, hypofibrinogenemia, and CD25soluble >2400 UI/mL) [3]. Our patient presented with a prolonged fever, splenomegaly, and adenopathies, with a biological analysis. In a study by Henderson and Cron [4], 8% of pediatric MAS cases were associated with malignant diseases, including Hodgkin lymphoma. The term MAS was first described by Claude Griscelli and his team in 1985 in seven patients with juvenile idiopathic arthritis. Thus, pediatric MAS secondary to Hodgkin lymphoma is a rare entity. Certain malignancies, particularly lymphoma, can be triggers for MAS, although this effect is less common in children than in adults. MAS present before the underlying malignancy is detected in the study by Sen ES et al. [5]. Such was the case of our patient, where the etiological workup of MAS ultimately revealed an underlying Hodgkin lymphoma. The clinical and laboratory features can also occur in the context of severe systemic infections such as sepsis. A negative infectious workup and the absence of improvement with anti-infective treatment support an inflammatory origin (MAS). Hyperferritinemia is a crucial criterion for the diagnosis of MAS. The bone marrow examination serves two purposes in the diagnostic process: to investigate bone marrow involvement, such as leukemia or other neoplasms, and to detect signs of MAS. It is essential to always consider MAS, as it can complicate various conditions, including infections, inflammatory diseases, and neoplasms.

EBV is a frequent cause of secondary MAS and a risk factor for Hodgkin lymphoma. In our case, EBV serology was requested as part of the etiological workup for prolonged fever. Therefore, EBV infection was confirmed. In a study by Flerlage et al., lymph-node histology with immunohistochemical complement for CD30, CD15, CD20, and CD3 was used to confirm the diagnosis of Hodgkin lymphoma [6]. All patients with Hodgkin lymphoma present with supradiaphragmatic lymphadenopathy, and 30% of patients present with systemic symptoms with extranodal site involvement [7]. The patient presented with fever, cough, and general deterioration of health without respiratory distress or seizures. EBV-induced MAS has a high mortality rate (41%) [8]. Moreover, EBV infection is implicated in 8-27.8% of malignancies associated with MAS cases in pediatric populations [9]. Therefore, EBV infection plays a key role in the onset of MAS and Hodgkin lymphoma. Our patient presented with two risk factors of MAS: EBV infection and Hodgkin lymphoma. Ataxia-telangiectasia, which is a rare and complex disease with an incidence of one in 40,000-200,000 population, is a risk factor for Hodgkin lymphoma. Diagnosis of Hodgkin lymphoma can be confirmed by cervical lymph node histology [10]. The treatment aimed to control both the hyperinflammatory syndrome and its underlying cause. Our patient was treated with intravenous corticosteroid therapy, resulting in favorable clinical improvement. The prognosis depends on the promptness of intervention and the underlying cause. The initiation of the chemotherapy protocol in the pediatric onco-hematology unit led to a favorable clinical, biological, and radiological response. 

## Conclusions

MAS is a rare mode of presentation of Hodgkin lymphoma in children. Early recognition of MAS can reduce morbidity and mortality rates. EBV infection and ataxia-telangiectasia are proven risk factors for lymphoma disease and MAS. Although it is difficult to distinguish MAS from severe infections, the persistence of symptoms despite well-conducted antibiotic treatment is a strong argument for MAS. Cervical lymphadenopathy, being accessible for biopsy, can facilitate the diagnosis of Hodgkin lymphoma. Corticosteroid therapy, combined with specific treatments (i.e., chemotherapy), helps to control the major inflammatory syndrome. Early treatment improves the prognosis of MAS. Large-scale studies are needed to better investigate the relationship between EBV, MAS, and Hodgkin lymphoma.
